# A Modified Sparrow Search Algorithm with Application in 3d Route Planning for UAV

**DOI:** 10.3390/s21041224

**Published:** 2021-02-09

**Authors:** Guiyun Liu, Cong Shu, Zhongwei Liang, Baihao Peng, Lefeng Cheng

**Affiliations:** 1 School of Mechanical and Electric Engineering, Guangzhou University, Guangzhou 510006, China; Liangzhongwei@gzhu.edu.cn (Z.L.); chenglefeng@gzhu.edu.cn (L.C.); 2 School of Electronics and Communication Engineering, Guangzhou University, Guangzhou 510006, China; 2111907071@e.gzhu.edu.cn (C.S.); 2111807063@e.gzhu.edu.cn (B.P.)

**Keywords:** unmanned aerial vehicle, optimization algorithm, modified sparrow search algorithm, route planning

## Abstract

The unmanned aerial vehicle (UAV) route planning problem mainly centralizes on the process of calculating the best route between the departure point and target point as well as avoiding obstructions on route to avoid collisions within a given flight area. A highly efficient route planning approach is required for this complex high dimensional optimization problem. However, many algorithms are infeasible or have low efficiency, particularly in the complex three-dimensional (3d) flight environment. In this paper, a modified sparrow search algorithm named CASSA has been presented to deal with this problem. Firstly, the 3d task space model and the UAV route planning cost functions are established, and the problem of route planning is transformed into a multi-dimensional function optimization problem. Secondly, the chaotic strategy is introduced to enhance the diversity of the population of the algorithm, and an adaptive inertia weight is used to balance the convergence rate and exploration capabilities of the algorithm. Finally, the Cauchy–Gaussian mutation strategy is adopted to enhance the capability of the algorithm to get rid of stagnation. The results of simulation demonstrate that the routes generated by CASSA are preferable to the sparrow search algorithm (SSA), particle swarm optimization (PSO), artificial bee colony (ABC), and whale optimization algorithm (WOA) under the identical environment, which means that CASSA is more efficient for solving UAV route planning problem when taking all kinds of constraints into consideration.

## 1. Introduction

### 1.1. Research Background

The three-dimensional (3d) route planning for unmanned aerial vehicle (UAV) can be considered as a multi-constraint global optimization problem [1], and the main purpose of this problem is to search for the optimal route from the departure point to the target point autonomously according to the task requirements and the flight constraints. In recent years, with the broad use of different kinds of UAV, this problem has drawn widespread attention from researchers. 

In recent years, various approaches have been put forward for solving this complex optimization problem, such as A* algorithm [2], rapidly exploring random trees [3], potential field based method (PFM) [4], genetic algorithm (GA) [5], linear programming [6], and artificial intelligence guidance [7]. These methods can be classified as deterministic algorithms and non-deterministic algorithms. Obviously, most of the practical engineering problems have many local optimal solutions [8,9]. Although deterministic algorithms are quite mature in mathematical theory, there also exists the problem that they are less effective in dealing with discontinuous and non-derivative functions, and they are easily trapped into local optimal solutions when solving the UAV route planning problem with multiple constraints [10]. Due to those problems that deterministic algorithms have, researchers have begun to pay more attention to heuristic algorithms. The most important feature of the heuristic algorithm is that it introduces a stochastic approach, which provides the opportunity to get rid of the local optimal solutions. Compared with classical methods, the heuristic algorithms require less computation due to their independence of initial conditions. In general, it is significant to use heuristic algorithms to obtain the optimal solution for global optimization problems.

### 1.2. Related Work

With the development of computer science, some heuristic algorithms have been widely applied to deal with UAV route planning problems. In Table 1, the recent relevant studies are shown as below.

Inspired by the predatory behavior of sparrows, the sparrow search algorithm (SSA) is firstly proposed in Ref. [27]. As a heuristic algorithm, the SSA imitates the predatory activity and the escape activity of a sparrow group, which has better convergence performance than some other swarm-intelligence algorithms. Because of its few controlling parameters as well as easy implementation, SSA has been successfully used in various practical engineering applications. For example, Ref. [28] proposed a power flow management of a hybrid renewable energy source (HRES) with SSA. Ref. [29] used an improved SSA to solve the renewable energy system optimization problem. In Ref. [30], the authors combined the neural networks with the SSA for the obstacle avoidance. In Ref. [31], the authors proposed an adaptive SSA to identify the optimal parameter of proton exchange membrane fuel cell stacks.

### 1.3. Contributions

In this paper, a novel modified sparrow search algorithm called CASSA is proposed for solving this complex optimization problem. The modified method includes the following steps: (1) The chaotic strategy is used to enhance the randomness of the positions of the initial population; (2) an adaptive inertia weight is applied to balance the convergence rate and exploration capabilities of the algorithm; (3) the Cauchy–Gaussian mutation strategy is adopted to improve the capability to get rid of stagnation. The effectiveness of the proposed modified algorithm is verified through the comparison among it and other algorithms, including original SSA, PSO, ABC, and WOA. Finally, the modified SSA is successfully applied to search the best route in the complicated 3d environment for UAV. The final experimental results evidenced that CASSA has the most excellent capability among all the algorithms tested in this paper.

The structure of this paper is shown as below. In Section 2, the original sparrow search algorithm is described in detail; Section 3 shows the motivation of improving CASSA and its details, including the chaotic strategy, the adaptive weight parameters, and the Cauchy–Gaussian mutation; Section 4 conducts comparison experiments on some classical benchmark functions and evaluates the effectiveness of each part of the proposed CASSA; Section 5 introduces the mathematical model of UAV route planning in detail and shows the experimental results of this problem; finally, Section 6 concludes with an integrated summary of this paper. 

## 2. Details of Optimization Techniques

### 2.1. Overview of Sparrow Search Algorithm

The original SSA is a heuristic algorithm motivated by the foraging and anti-predation behaviors of sparrow colony. This algorithm was put forward by Ref. [27] in 2020 and has drawn great attention recently. During the foraging process of sparrows, the colony is divided into finders and entrants. The finders have better fitness values and provide foraging areas and directions for the entire sparrow colony, while the entrants use the position of finders to get food. When the sparrow colony detects the danger and the alarm value exceeds the safety value, the sparrows will act against predation. The framework of original SSA is composed of the following three main parts.

#### 2.1.1. Updating Finder Location

In the search process, the finders with better fitness values are given priority for getting food. As the finders have the responsibility to search for food and direct the movement of the whole population, they can find food in a broader range compared with other sparrows. During each iteration, the location of the finder is updated as below:

(1) $$\begin{array}{ll} X_{i}^{t+1}=\left\{\begin{array}{cc} X_{i}^{t}\times exp\left(\frac{-i}{a\cdot T_{max}}\right), & R_{2}<ST\\ X_{i}^{t}+Q\cdot L, & R_{2}\geq ST \end{array}\right.\\ with & \\ X={\left[X_{1},X_{2}\cdots X_{i}\cdots X_{n}\right]}^{T},X_{i}=[X_{i,1},X_{i,2}\cdots X_{i,d}] \end{array},$$

where t represents the current iteration; n is the number of sparrows; d denotes the dimension of the variables; $T_{\max}$ is the largest number of the iteration; $X_{i}^{t}$ represents the position of the *i*th individual at iteration *t*; α∈(0,1] denotes a random number; $R_{2}\in (0,1]$ represents the alarm value and ST∈[0.5,1) represents the safety threshold; L is a 1×d matrix that each factor inside is 1; Q is a random number that obeys the normal distribution with mean 0 and variance 1; if $R_{2}<ST$, it denotes that the foraging environment is safe, while $R_{2}\geq ST$ signifies that some individuals have already encountered predators and therefore all sparrows need to fly quickly to other safe areas.

**Figure 1:**
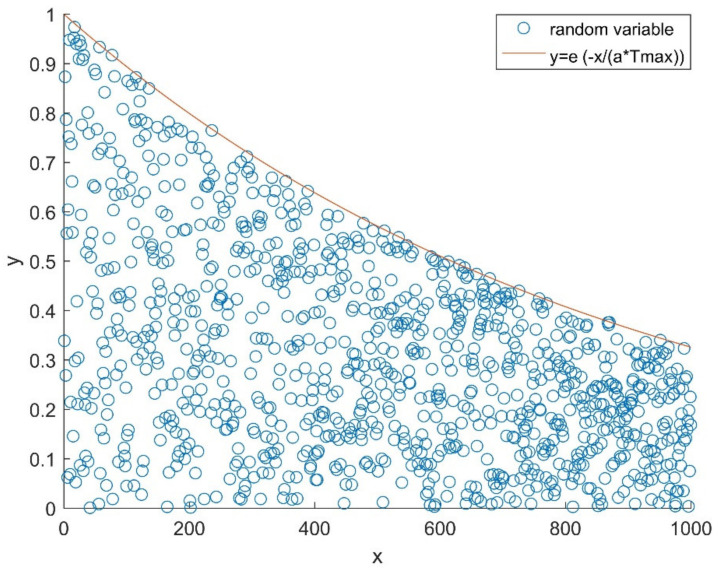
Distribution of the random variables between 0 and *y*.

According to Equation (1), it can be seen that when $R_{2}<ST$, the next generation of finder will move around the current position. Equation (2) reveals the variation in the range of values of the finder’s position.

(2) $$y=exp\left(\frac{-x}{a\cdot T_{max}}\right),$$

where x represents the iteration times, and y represents the range of value variation for finder’s position. Figure 1 shows the distribution of a random value between 0 and *y*, which represents the change of the updated range of the finder’s position when the foraging environment is safe. As x becomes larger, y gradually narrows slowly from (0,1) to approximately (0,0.3). When x is smaller, the probability of y taking on a value close to 1 is higher, and as x increases, the distribution of the values of y becomes more even. Therefore, when $R_{2}<ST$, the range of value variation of each dimension of the sparrow is getting smaller. This search strategy makes the SSA extremely capable of local search, but it also leads to a tendency to fall into the local optimal solutions in the late iterations. 

#### 2.1.2. Updating Entrant Location

The rest of the sparrow colony are entrants, which monitor the finders frequently. As soon as they notice that the finder has found better food, they will quit their current position and fly to better foraging areas. The location of the entrants is updated as below:

(3) $$X_{i}^{t+1}=\left\{\begin{array}{cc} Q\cdot \exp\left(\frac{X_{worst}-X_{i}^{t}}{\alpha \cdot T_{\max}}\right), & i>\frac{n}{2}\\ X_{best}^{t+1}+\left|X_{i}^{t}-X_{best}^{t+1}\right|\cdot A\cdot L, & i\leq \frac{n}{2} \end{array}\right.,$$

where $X_{best}$ denotes the elite individual position, i.e., the current best position; $X_{worst}$ is the current global worst position; A denotes a d×d matrix for which each factor inside is assigned 1 or −1 in random. When i≤n/2, it suggests that the *i*th entrant is foraging near the best location, if i>n/2, it means that the *i*th entrant with the worse fitness is needed to fly to another place for food. 

#### 2.1.3. Detection and Early Warning Behavior

In the colony, all sparrows have a scouting and early warning mechanism. Generally, the sparrows that are aware of the predator account for 15% to 30% of the colony. The mathematical model can be described as below:

(4) $$X_{i}^{t+1}=\left\{\begin{array}{cc} X_{best}^{t}+\beta \left|X_{i}^{t}-X_{best}^{t}\right|, & f_{i}>f_{b}\\ X_{best}^{t+1}+K\frac{\left|X_{i}^{t}-X_{best}^{t+1}\right|}{(f_{i}-f_{w})+\varepsilon }, & f_{i}=f_{b} \end{array}\right.,$$

where β represents the random step length control coefficient, which obeys the normal distribution with variance of 1 and mean value of 0; K∈[−1,1] is a random number; $f_{i}$ denotes the fitness value of the *i*th individual; $f_{b}$ denotes the current global best fitness; $f_{w}$ denotes the current global worst fitness; ε represents a smallest parameter to avoid the zero-division-error. When $f_{i}>f_{b}$, it means that the individual is at the edge of the colony. If $f_{i}=f_{b}$, this indicates that the sparrows in the middle of the colony are aware of the danger and need to fly closer to the safe place. 

### 2.2. Proposed Modified Sparrow Search Algorithm (CASSA)

According to the results of previous research, the original SSA has better robustness and rapid convergence speed [27,28,29,30,31]. However, some shortcomings still exist in SSA, such as being easily trapped into local optimal solutions and lower solution precision. The initialization strategy of SSA is a simple random method, which makes the performance of the algorithm largely depend on the diversity of the initialized populations. Additionally, in the late iterations of the algorithm, the sparrow group gradually clusters around the found optimal position, making it easily get trapped into local optimal solutions. Therefore, in order to further enhance the SSA’s capability, some specialized strategies are adopted. The detailed definitions of CASSA are shown as the followings.

#### 2.2.1. Chaotic Strategy 

In solving complex optimization problems, SSA has the disadvantage of poor population diversity in the late iterations. Recently, chaotic sequences [32] have been applied to the intelligence algorithms in many optimization applications. For instance, in Ref. [33], chaotic sequences are applied to dynamically improve population size to avoid immature convergence; in Ref. [34], chaotic sequences are used in the generation of the initial population and the performance of the mutation operators. In this paper, chaotic sequences are used to improve the population diversity of SSA. Chaotic sequences can map by different chaotic models such as the Tent map, Logistic map, Kent map, and Cubic map. Ref. [35] demonstrates that the Cubic map has better uniformity than others. Therefore, the cubic mapping chaos sequences are adopted for the generation of CASSA population. The diversity of the CASSA’s population is improved by the ergodicity and the initial sensitivity of the chaotic maps. The mathematical formula is described as below:

(5) $$X_{i}=X_{lb}+\frac{\left(X_{ub}-X_{lb})\times (y_{i}+1\right)}{2},$$

(6) $$\begin{array}{c} y_{i+1}=4y_{i}{}^{3}-3y_{i}\\ -1<y_{i}<1,\;y_{i}\neq 0,\;i=0,1,\ldots ,N \end{array},$$

where $X_{i}$ represents the individual variable values of sparrows; $X_{lb}$ and $X_{ub}$ correspond to the upper and lower bounds in the solution space, respectively; N represents the population size. Firstly, let D denote the dimension, and a D-dimensional vector with values of [−1,1] in each dimension is randomly generated as the first operator. Then Equation (5) is used to iterate over each dimension of the first operator to obtain the remaining (N−1) operators. Finally, Equation (6) is used to map the values of the operators generated by the cubic mapping onto individual of sparrows.

#### 2.2.2. Adaptive Inertia Weight Strategy

**Figure 2:**
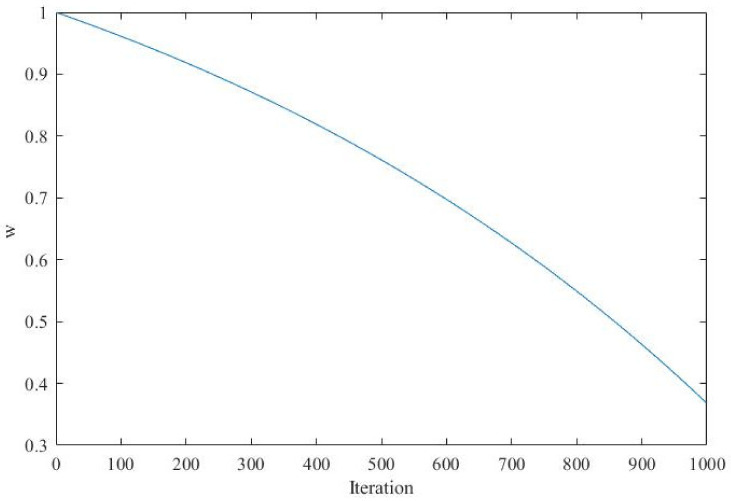
The changing trend of *w*.

Whether the SSA can find the optimal solution is largely determined by the search ability of the finder. The locations of individuals in the searching range are distributed at random. When there are no adjacent sparrows near the current finder, a random search strategy will be conducted. It should be noticed that this mode not only slows down the convergence speed, but also decreases the convergence accuracy under the limited number of iterations. For further improvement of the finder’s performance, an adaptive inertia weight is introduced to Equation (7). The mathematical formula is described as below:

(7) $$X_{i}^{t+1}=\left\{\begin{array}{cc} X_{i}^{t}\cdot \exp\left(\frac{-i}{w\cdot \alpha \cdot T_{\max}}\right), & R_{2}<ST\\ X_{i}^{t}+Q\cdot L, & R_{2}\geq ST \end{array}\right.,$$

(8) $$w=2-\frac{\exp(t_{}/T_{\max})-1}{\exp(1)-1},$$

where t is the current iteration times; $T_{\max}$ represents the maximum iteration times; w is an inertia weight that adaptively decreases as the number of iterations increases. By introducing this inertia weight, the range of values taken by α is adaptively controlled. As is shown in Figure 2, at the beginning of the iterations, a larger value of w gives the algorithm a larger range of optimization. At the end of the iterations, a smaller value of w is conducive to enhancing the convergence accuracy of the algorithm.

#### 2.2.3. Cauchy–Gaussian Mutation Strategy

In the late iterations of SSA, sparrows gradually move closer to the optimal individuals, which leads to a lack of population diversity and a tendency for the algorithm to converge prematurely. For solving this problem, the Cauchy–Gaussian mutation strategy [36] is introduced in this paper. The individual with the best current fitness is selected for mutation. Then, its positions before and after mutation are compared, after which the better position is chosen to enter the next iteration. The mathematical definition of the Cauchy–Gaussian mutation strategy is described as below:

(9) $$U_{best}^{t+1}=X_{best}^{t}[1+\lambda_{1}cauchy(0,\sigma^{2})+\lambda_{2}Gauss(0,\sigma^{2})],$$

(10) $$\sigma =\left\{\begin{array}{cc} 1, & f(X_{best})<f(X_{i})\\ \exp\left(\frac{f(X_{best})-f(X_{i})}{\left|f(X_{best})\right|}\right), & otherwise \end{array}\right.,$$

where $X_{best}$ is the elite individual position, $U_{i}^{t+1}$ represents the position of the elite individual after mutation, $\sigma^{2}$ represents the standard deviation of the Cauchy–Gaussian mutation strategy. $cauchy(0,\sigma^{2})$ is a random variate satisfying Cauchy distribution, and $Gauss(0,\sigma^{2})$ is a random variate satisfying Gaussian distribution. $\lambda_{1}=1-t^{2}/T_{{}_{\max}}^{2}$ and $\lambda_{2}=t^{2}/T_{{}_{\max}}^{2}$ are the dynamic parameters that are adaptively adjusted with the times of iteration. In Equation (9), $\lambda_{1}$ is larger at the initial stage so that the algorithm can explore the optimal solution in a larger range with a larger mutation step. $\lambda_{2}$ has a small mutation step, which facilitates the algorithm to search near the optimal solution. During the search process, $\lambda_{1}$ decreases gradually, while $\lambda_{2}$ keeps increasing. 

**Figure 3:**
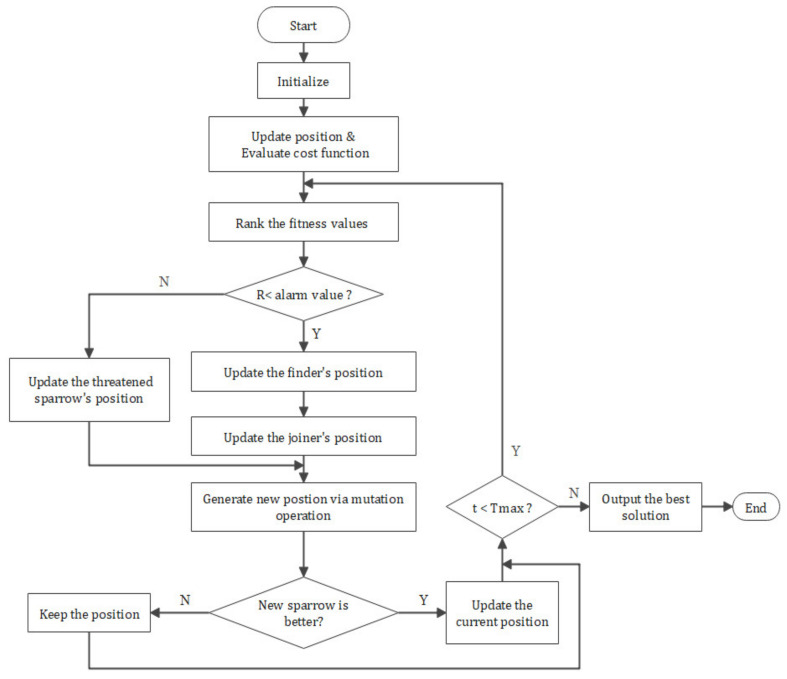
The flowchart of CASSA.

The flowchart is shown in Figure 3, and the detailed implementation procedure of CASSA is illustrated in Algorithm 1.
**Algorithm 1** The framework of CASSA/*Initialization*/ 1. Set the maximum iterations as $T_{\max}$; 2. Set the number of finders as $F_{d}$; 3. Set the number of threatened sparrows as $S_{d}$; 4. Set the alarm value as G; 5. Set the number of sparrows as n; 6. Initialize the position of n sparrows using Equation (5); /*Iterative search*/ 7. **while** ($t<T_{\max}$) 8. Rank the fitness values and find the best individual and the worst individual currently; 9.  G=rand(1); 10. **for**
$i=1:F_{d}$ 11.   Update the finder’s position using Equation (7); 12. **end for** 13. **for**
$i=(F_{d}+1):n$ 14.   Update the entrant’s position using Equation (3); 15. **end for** 16. **for**
$i=1:S_{d}$ 17.   Update the threatened sparrow’s position using Equation (4); 18. **end for** 19. Select the top S elite individuals and implement adaptive mutation for them by Equation (9); 20. Get the current new position; 21. If the new position is better than before, update it; 22. t=t+1; 23. **end while** 24. Output the best solution

## 3. Experiment for Benchmark Functions

**Figure 4:**
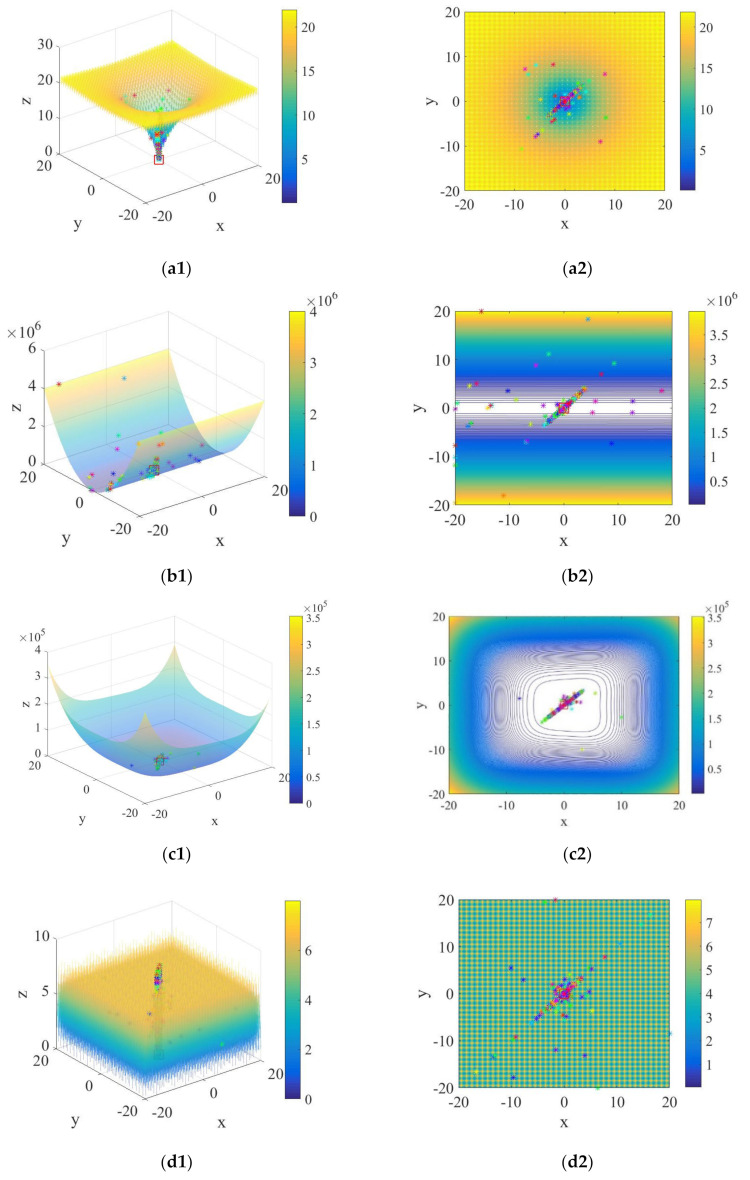
The trajectories of CASSA on the 3-D version of the four test functions: (**a1**,**a2**) Ackley function; (**b1**,**b2**) Cigar function; (**c1**,**c2**) Schwefel function; (**d1**,**d2**) Weierstrass function.

**Figure 5:**
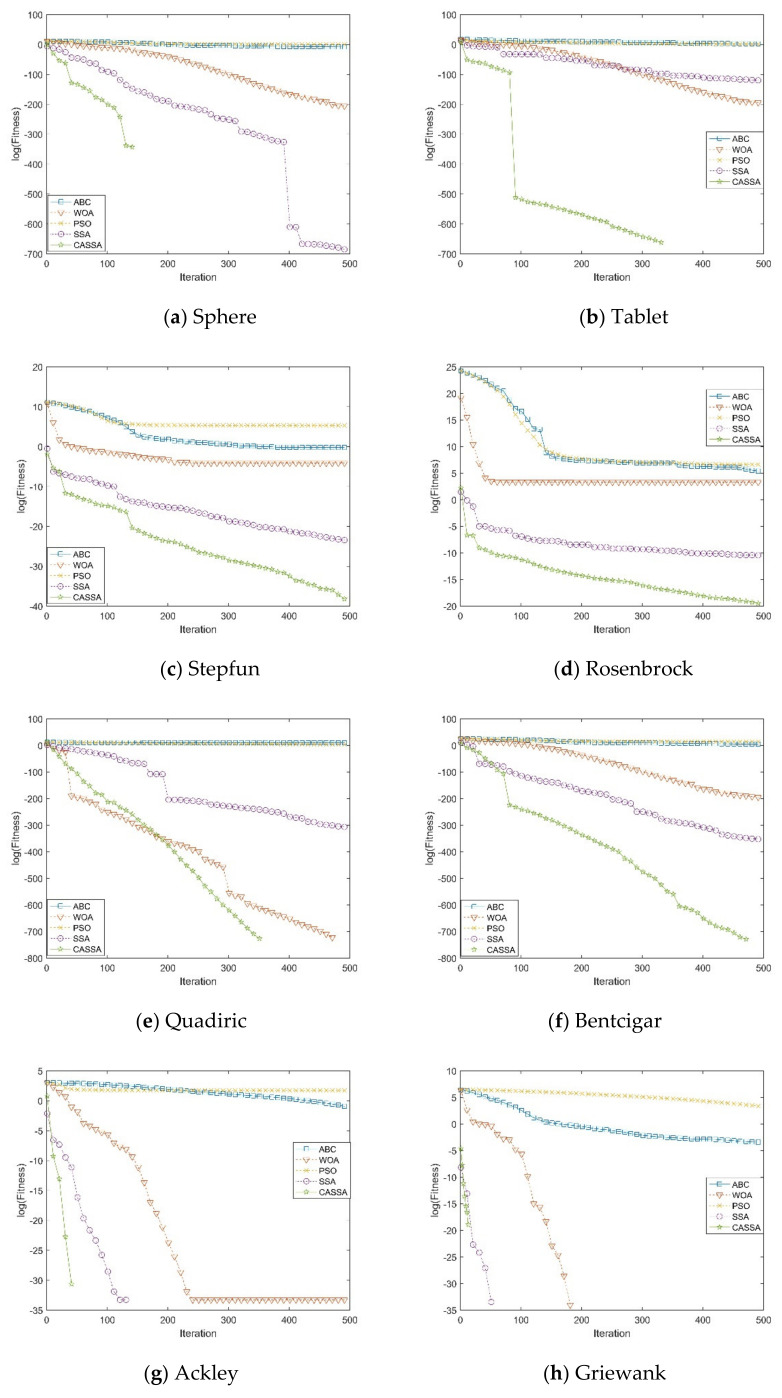

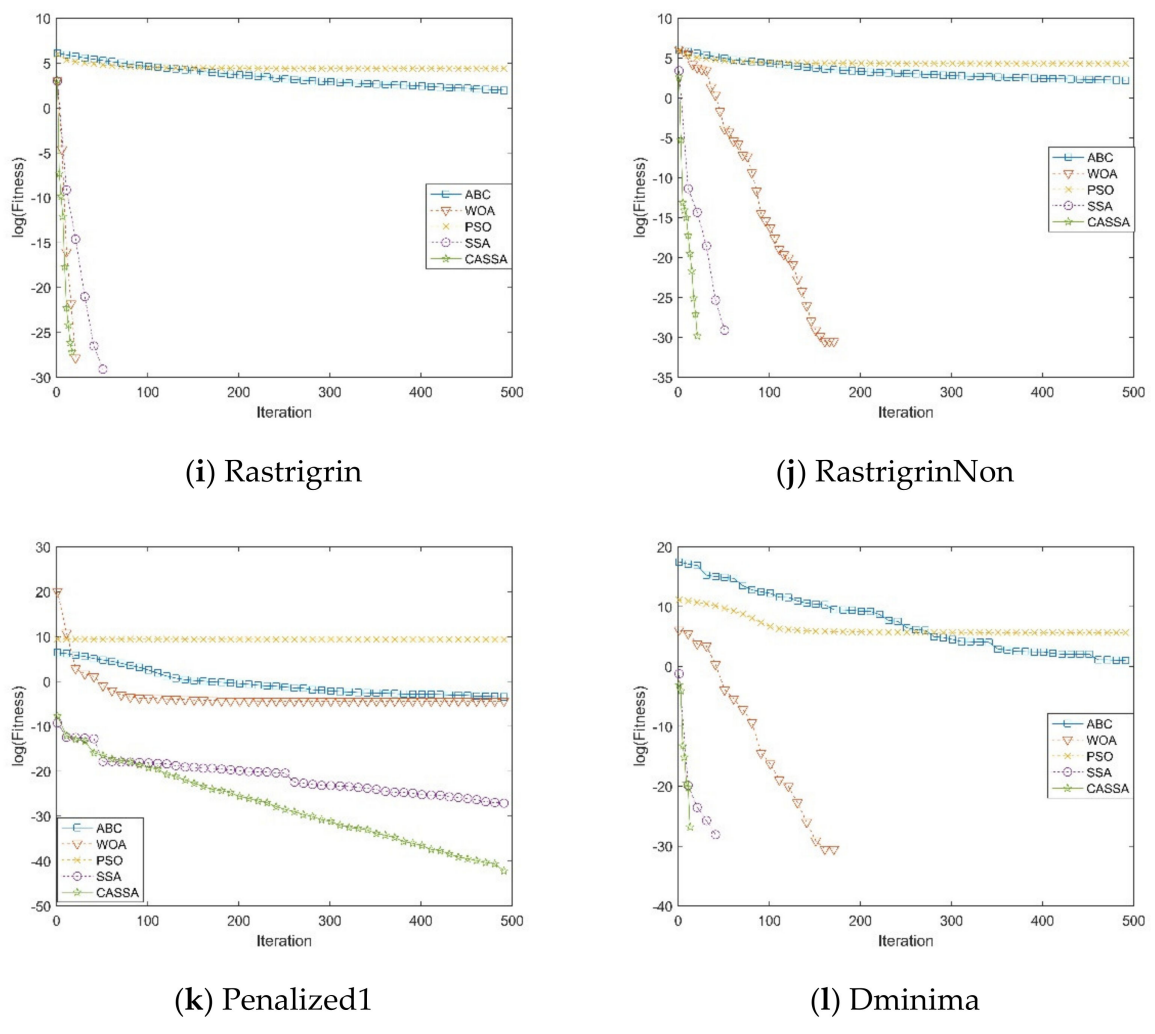
Convergence curve of five algorithms on twelve benchmarks functions.

In this section, there are four tested functions shown in Table 2, and the trajectories of individuals of CASSA in these test functions are presented in Figure 4. It shows that most individuals move towards the global optimum in unimodal functions. However, in the more complex multi-modal functions, although many individuals stagnate at the local minimums, some individuals can still avoid them and aggregate towards the global optimum. To verify whether it is feasible and highly efficient, the proposed CASSA is compared with four algorithms in the experiments, including original SSA, PSO, ABC, and WOA. There are twelve classical benchmark functions [37] shown in Table 3, which consists of the information of their formula, initialization range, and global optimum. Among these benchmark functions, f1∼f6 are unimodal functions, and f7∼f12 are multi-modal functions. In all cases, we conducted the independent trials 30 times on each benchmark function. The maximum number of iterations is 500, and the population size is 50 in each experiment. Finally, we obtained the best value, mean value, and the standard deviation (Std.) of the objective function values. With the same benchmark function, the best value and average value denote the exploration ability and convergence accuracy, respectively, and the standard deviation denotes the stability of the algorithm. The experimental results are shown in Figure 5 and Table 4.

As is shown in Figure 5, in the experiments on unimodal functions, CASSA can quickly find the optimal values of f1, f2, f5, and f6. Although the optimal values of f3 and f4 are not found, CASSA still has the fastest convergence speed and accuracy among all the tested algorithms. In the experiments on the multi-modal functions, only CASSA and SSA can find the optimal values of f7. For f8, f9, f10, and f12, CASSA, SSA, and WOA can find the optimal values, but CASSA requires the smallest iteration times. For f11, all algorithms fail to find the optimal value, but CASSA still finds the near-optimal solution with the highest accuracy.

In terms of convergence value and convergence speed, CASSA is better than the other four test algorithms. As is shown in Table 4, the test results of unimodal functions and multi-modal functions show that the mean and variance of CASSA are much smaller than those of the other four algorithms, which shows that the optimal values obtained by the algorithm are highly stable and robust and can be effectively implemented to avoid premature convergence.

With the purpose of further assessing the performance of these heuristic algorithms and proving a significant improvement of CASSA compared with the other four algorithms, a non-parametric statistical test is performed with the Wilcoxon [38] rank sum test set at a significance level of α = 5%. It is generally considered that when *p* < 5%, it is significantly different, while when *p* ≥ 5%, it is not significantly different. As is shown in Table 5, *p* of CASSA are all less than 5%, which means that compared with the search ability of SSA, that of CASSA has been greatly improved. Additionally, the optimization accuracy and robustness of CASSA are better than other popular intelligent algorithms.

## 4. UAV Route Planning Strategy

In this section, the application of CASSA to 3d UAV route planning will be discussed in detail. The UAV route planning aims to minimize the cost function in the task space, which can be defined as an optimization problem with multi-constraint. Generally, the UAV route generated by meta-heuristic algorithms is composed of the line segments, which is unsuitable for exact flight. To solve this problem, some methods are used to further optimize the route generated, such as the B-spline curve [39], the Bezier curve [40], and the simple circular arcs [41]. The B-spline curve has the unique superiority in many ways, such as locality, geometrical invariability, symmetry, recursion, continuity, convex hull characters, and convexity-preservation. It is highly appropriate to use the B-Spline curves in the process of optimization, because merely a few variables are required to define the complex curved routes. Therefore, in this paper, the B-Spline curves smoothing strategy is used to smooth the generated route. With the given departure point and target point, some control points in the map space are set and applied to generate the B-Spline curve. The points on the B-Spline curve serve as the waypoints, of which the coordinates can evaluate the cost of the UAV route.

### 4.1. B-Spline Curve

The construction of B-Spline curve is made up of blending functions, which denotes that B-Spline curve is a parametrized curve. In a whole route, the number of waypoints is n, the number of control points of the corresponding curve is n + 1, with the coordinates $\left(x_{0},y_{0},z_{0}),\ldots ,(x_{p},y_{p},z_{p}),\ldots ,(x_{n},y_{n},z_{n}\right)$; then, the coordinates $\left(x_{e},y_{e},z_{e}\right)$ of the B-Spline curve can be defined as follows:

(11) $$\begin{array}{ll} & \left\{\begin{array}{c} x_{e}=\sum_{i=1}^{n}x_{i}\times C_{i,p}(e)\\ y_{e}=\sum_{i=1}^{n}y_{i}\times C_{i,p}(e)\\ z_{e}=\sum_{i=1}^{n}z_{i}\times C_{i,p}(e) \end{array}\right.\\ & with\\ e_{i}= & \left\{\begin{array}{ll} 0, & \;i<p+1\\ i-p, & \;p+1\leq i\leq n\\ n-p+1, & \;n<i \end{array}\right. \end{array},$$

where p represents the order of the curve, which influences the smoothness of the B-Spline curve; the free parameter e varies from 0 to n−p+1, which generates a series of discrete points. The blending functions are designed by a knot vector $E=\left\{e_{0},\ldots ,e_{m}\right\}$ recursively as follows:

(12) $$\left\{\begin{array}{l} C_{i,p}=\frac{e-e_{i}}{e_{i+p}-e_{i}}C_{i,p-1}(e)+\frac{e_{i+p-1}-e}{e_{i+p+1}-e_{i+1}}C_{i+1,p-1}(e)\\ C_{i,0}=\left\{\begin{array}{c} 1,{}^{}e_{i}\leq e\leq e_{i+1}\\ 0{,}^{}{{}^{}}^{}otherwise \end{array}\right. \end{array}\right.,$$

**Figure 6:**
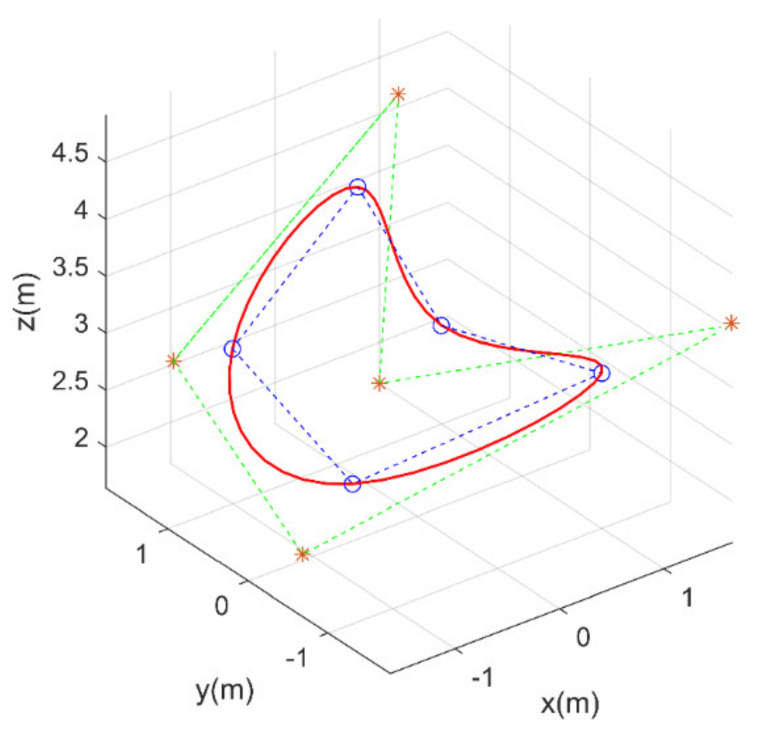
A 3d cubic (k = 3) B-Spline curve.

The B-Spline curve is used to determine the flight route, and it has the advantage of illustrating a complex non-monotonic 3d curve with controlled smoothness through a few designed parameters. Figure 6 shows a 3d cubic B-Spline curve (k = 3) with its control points and the corresponding control polygon (the green line). After this procedure, the original route (the blue line) could be replaced by the new smooth route (the red curve).

### 4.2. Cost Function

The problem of UAV route planning is defined by a series of optimization criteria and constraints, which consist of the minimization of the threats of damage of the UAV, the constraints imposed by the UAV’s dynamic property and the flight environment. The cost function is described as the total of three functions, given as follows:

(13) $$J_{\cos t}=w_{1}J_{path}+w_{2}J_{height}+w_{3}J_{turn},$$

where $J_{\cos t}$ represents the total cost function; $J_{path}$ denotes the length cost of the route of UAV; $J_{height}$ denotes the height cost of UAV; $J_{turn}$ denotes the smoothness cost of the planning route; $w_{i}$ is the weights of the above functions, which are given as follows:

(14) $$\left\{\begin{array}{l} w_{i}\geq 0\\ \sum_{i=1}^{3}w_{i}=1 \end{array}\right.,$$

In the process of UAV route planning, it is obvious that the shorter route needs less time and less fuel consumption for flight. In the meanwhile, it is less likely to encounter an unknown danger. The route length is calculated with the following expressions:

(15) $$\begin{array}{l} J_{path}=\left\{\begin{array}{c} \infty ,\;passtheobstacles\\ \sum_{i=1}^{n-1}l_{i},\;otherwise \end{array}\right.\\ with\\ l_{i}={\left\|\left(x_{i+1},y_{i+1},z_{i+1})-(x_{i},y_{i},z_{i}\right)\right\|}_{2} \end{array},$$

where $\left(x_{i},y_{i},z_{i}\right)$ denotes the $i^{th}$ waypoint within the whole UAV route. If the line segment between every two waypoints passes the obstacles, the cost of the route needs to be processed by the penalty function. However, it is hard to express the infinite function in the practical experiment. Therefore, a large value, like 10^7^, can be added to solve this problem in the simulation.

In the meantime, the suitable flying height also has an important impact on the UAV route planning process. For most kinds of UAV, the flying height should not be changed too frequently and drastically. The stable flying height can save more fuel and ensure more security. Additionally, when UAV flies at a low height, it can benefit from the terrain mask effect, which can help to avoid unknown threats [42]. Therefore, the cost function of height is described as below:

(16) $$\left\{\begin{array}{c} H_{height}=\sqrt{\frac{1}{n}\sum_{i=0}^{n-1}{\left(z_{i}-\bar{z}\right)}^{2}}\\ \bar{z}=\frac{1}{n}\sum_{i=0}^{n-1}z_{i} \end{array}\right.,$$

The stability and maneuverability of UAV are constrained by the maximum turning angle at each waypoint. In the process of UAV route planning, the maximum turning angle should not be bigger than the maximum preset angle. Furthermore, when the UAV flight efficiency is considered, it is obvious that the UAV work condition will be inefficient when UAV does the turning maneuvers. The cost function of the turning angle can be defined as below:

(17) $$\begin{array}{l} J_{turn}=\left\{\begin{array}{c} \sum_{i=i}^{n}\left(\cos\varphi -\cos\theta_{i}\right){,}^{}\varphi \geq \theta_{i}\\ \infty {,}^{}{\begin{array}{cc} \begin{array}{cc} \begin{array}{cc} {{}^{}}^{} & \end{array} & \end{array} & \end{array}}^{}{{}^{}}^{}\varphi <\theta_{i} \end{array}\right.\\ with\\ \cos\theta =\frac{a_{i}^{T}a_{i+1}}{\left|a_{i}\right|\left|a_{i+1}\right|} \end{array},$$

where φ denotes the maximum angle; θ denotes the current angle; and $a_{i}$ represents a vector of the *i*th part of the whole route.

### 4.3. CASSA for 3d UAV Route Planning

**Figure 7:**
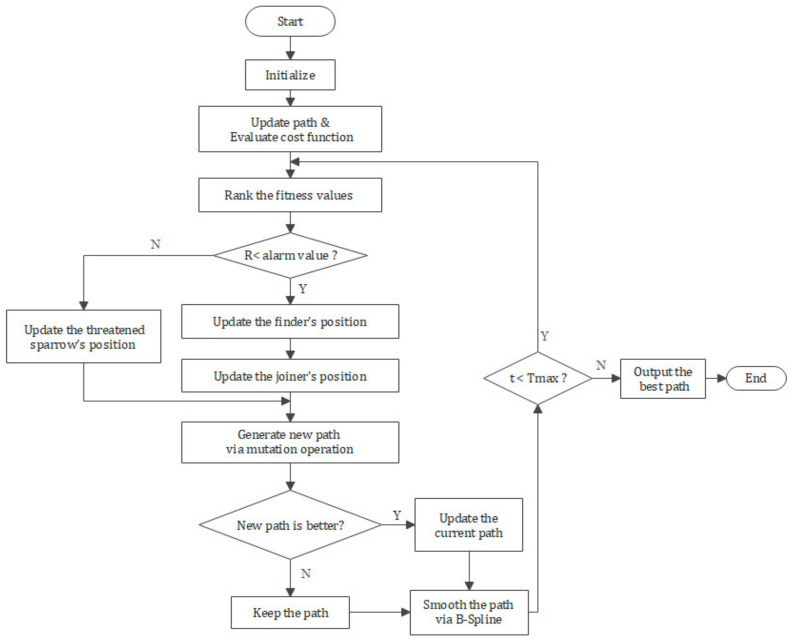
Flowchart of CASSA for 3d UAV route planning.

After the above discussion, the detailed implementation procedure of CASSA for UAV three-dimensional route planning is illustrated in Algorithm 2, and the flowchart is shown in Figure 7.
**Algorithm** **2** CASSA for 3d UAV route planning/*Initialization*/ 1. Set the parameters of CASSA same as Algorithm 1; 2. Set the start point $\left(x_{S},y_{S}\right)$, target point $\left(x_{T},y_{T}\right)$, the boundaries of the map space, and     the number of control points n; 3. Set the position$\left(x_{threat},y_{threat}\right)$ and the range of threats; /*Iterative search*/ 4. **while** ($t<T_{\max}$) 5. Each sparrow represents a route; sort the population of sparrows from best to worst by    order of cost function Equation (10) for each sparrow 6. G=rand(1); 7. **for**
$i=1:F_{d}$ 8.   Update the finder’s position using Equation (7); 9. **end for** 10. **for**
$i=(F_{d}+1):n$ 11.   Update the entrant’s position using Equation (3); 12. **end for** 13. **for**
$i=1:S_{d}$ 14.   Update the threatened sparrow’s position using Equation (4); 15. **end for** 16. Evaluate the cost for each route by Equation (13), then select the top S elite sparrows with the    best fitness value and implement adaptive mutation for them by Equation (9); 17. Get the current new route; 18. If the new route is better than before, update it; 19. Generate the B-Spline curve with the n waypoints. 20. t=t+1; 21. **end while** 22. Output the best route and its cost value. 23. **Post-process results and visualization**

## 5. Simulation Experiment

In this section, the capability of CASSA in solving the 3d UAV route planning problem will be assessed by a series of computational simulations. To conduct a fair comparison, all the experiments are performed on the PC with Intel (R) Core (TM) i5-9400 @2.40 GHz CPU and 16 GB RAM, and the simulations are compiled using Matlab-2016b under Win-10 platform. 

### 5.1. Experimental Parameters

**Figure 8:**
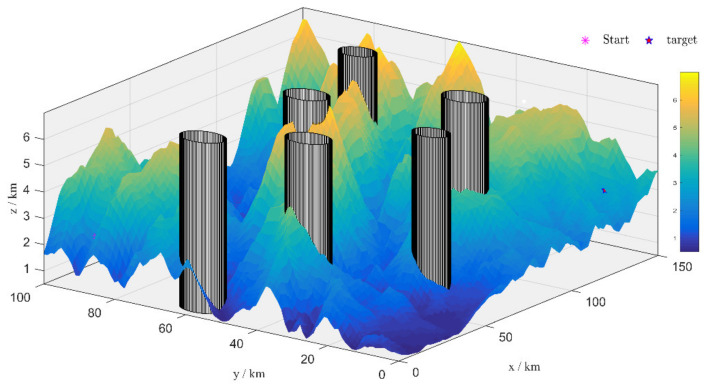
The scene of the 3d UAV route planning space.

The UAV mission region is [0, 150] km long, [0, 100] km wide, and [0, 8] km high, and UAV knows the terrain and threat’s position. The terrain is modeled by a processing elevation map of real terrain, while the threats are modeled as black columns. The coordinates value of the start point is (10.0,90.0,2.3 km), the coordinates value of the target point is (140.0,10.0,2.9 km). The 3d UAV route planning environment is shown in Figure 8.

There are five algorithms in the experiments, including CASSA, SSA, PSO, ABC, and WOA. In all experiments, the parameters of those algorithms are set according to their references respectively. To make a fair comparison, the maximum iterations of those algorithms is set as 200, while the population size is set as 50. For CASSA and SSA, using the same parameters setting is necessary, which are the number of the finders (Fd = 0.2), the number of the threatened sparrows (Sd = 0.15), and the alarm value (G = 0.8). In consideration of the randomness nature of heuristic algorithms, each tested algorithm is executed 30 times independently, and the experimental results are used for the evaluation and comparison of their performance.

### 5.2. Analysis of Experimental Results

**Figure 9:**
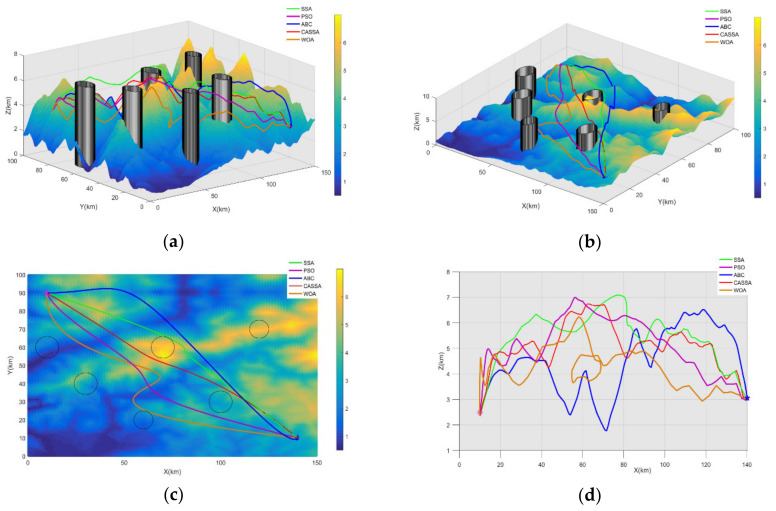
The best route of each algorithm. (**a**,**b**) Route comparison in three-dimensional space; (**c**) overhead view of Route comparison in three-dimensional space; (**d**) side view of route comparison.

**Figure 10:**
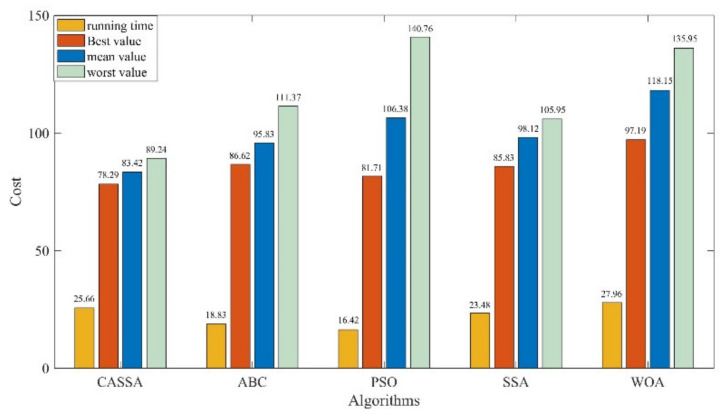
The statistical results of CASSA, ABC, PSO, SSA, and WOA.

**Figure 11:**
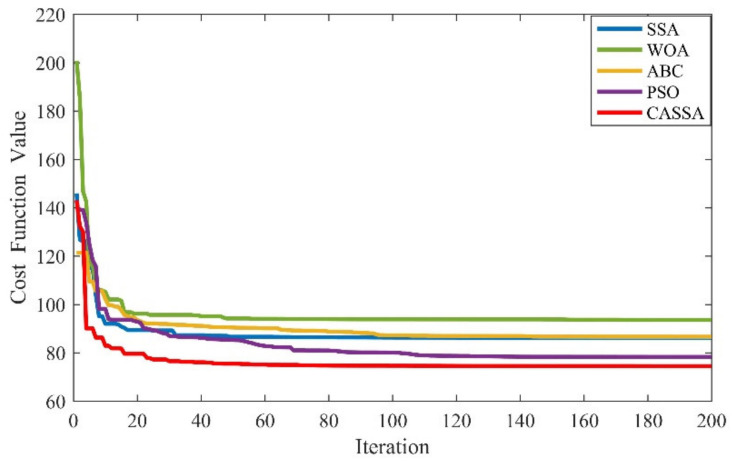
The best results of CASSA, ABC, PSO, SSA, and WOA.

For the objective of comparison, the best UAV routes generated by CASSA, SSA, PSO, ABC, and WOA during 30 independent runs are displayed in Figure 9, and the statistic results are showed in Figure 10. To show the difference between the tested algorithms intuitively, the best cost curves for each algorithm are displayed in Figure 11.

As the test results reveal, all the five tested algorithms found the safe flight route for UAV, but they have different performance on route length, flight altitude, and route smoothness. As experimental results display in Figure 9, it is apparent that among all the five algorithms, CASSA can find the best route for the UAV. The route generated by CASSA has a more suitable altitude, which can help UAV to benefit more from the terrain mask, and its length and smooth criteria are also better than other four algorithms. In Figure 10, it can be seen that CASSA has the best performance on the best cost value, mean cost value, and worst cost value, which shows that CASSA is superior to other four algorithms with regard to searching ability and stability. Among all the five algorithms, PSO takes the shortest running time and WOA takes the longest running time. The running time of CASSA is a little longer than SSA, mainly due to the variation operations added during the iteration of the algorithm. For offline route planning problems, the accuracy of the algorithm is more important than the running time, so the time taken to run CASSA is acceptable. In Figure 11, it should be noticed that CASSA not only has the best convergence value, but also has a good initial solution and the fastest convergence speed.

The 3d UAV route planning problem has many local solutions, making it challenging to solve by classical optimization algorithms. The reason why CASSA performed excellently on this complex optimization problem is due to the integration of the adaptive inertia weight and the Cauchy–Gaussian mutation operator. In the searching process, the adaptive inertial weight can control the search ability and convergence speed of CASSA, and the integration of the Cauchy–Gaussian mutation operator can avoid the local optimal solutions by suddenly changing the location of the elite sparrows in the search space. Therefore, the excellent exploration ability and local minimums avoidance of the proposed CASSA helps it to improve the drawbacks and surpass the original SSA. 

## 6. Conclusion and Future Work

This paper proposed a modified sparrow search algorithm called CASSA and successfully applied it for solving the 3d UAV route planning problem in complex task space. In the proposed CASSA, the chaotic strategy is used to enhance the stability of the algorithm, and an adaptive inertia weight is used to balance the convergence speed and exploration ability. The original SSA usually suffers from local optimal stagnation, but the Cauchy–Gaussian mutation operator integrated is able to help CASSA avoid this drawback by suddenly changing the position of the elite sparrows in the search space. Those modified strategies lead to the better performance of CASSA on the 3d UAV route planning problem. In order to make the route more viable for practical UAV flight mission, the B-Spline curve is used for smoothing the route generated by CASSA. The experimental results demonstrate that CASSA is an efficient and viable method in the field of UAV route planning.

There are many challenges for future work. For example, more constraints can be introduced, such as flight speed constraint, different types of obstacles, and dynamic threats. Moreover, it would be meaningful to apply CASSA for solving other optimization problems in the field of UAV, such as task assignment and formation control. 

## Figures and Tables

**Table 1:** Heuristic algorithms for UAV route planning.

Authors	Algorithms	Modified Strategy
Shin, J. et al. [11]	Improved particle swarm optimization (PSO) algorithm	Proposed the multiple balance strategy
Cekmez, U. et al. [12]	Multi ant colony optimization (MACO) algorithm	Proposed the new information exchange strategy
Li, B. et al. [13]	Improved artificial bee colony (ABC) Algorithm	Proposed the balanced evolution strategy
Pan, J. et al. [14]	Chaotic cuckoo search (CCS) algorithm	Integrated the chaotic strategy into CS
Pandey, P. et al. [15]	Improved glowworm swarm optimization (GSO) algorithm	Introduced the genetic operators of mutation and crossover into GSO
YongBo, C. et al. [16]	Modified wolf pack search (WPS) algorithm	Introduced the mutation operators into WPS
GaiGe, W. et al. [17]	Improved bat algorithm (IBA)	Combined the BA with Differential Evolution (DE)
Wu, J. et al. [18]	Improved whale optimization algorithm (IWOA)	Proposed an adaptive chaos–Gaussian switching strategy
ChenZhi, Q. et al. [19]	Hybrid grey wolf optimizer (GWO) (HSGWO-MSOS)	Combined the simplified GWO and modified symbiotic organisms search (MSOS)
Jize, L. et al. [20]	Modified PSO algorithm	Introduced the genetic algorithm and chaos theory into PSO
Pierre, D.M. et al. [21]	Master–slave parallel vector-evaluated genetic algorithm (MSPVEGA)	Proposed the Master–slave parallel vector-evaluated strategy
Xinhua, W. et al. [22]	Improved ant colony algorithm (ACA)	Proposed a new node selection strategy
Sun, Y. et al. [23]	Modified clustering algorithm (CA)	Combined the improved clustering algorithm and ant colony algorithm
Yubing, W. et al. [24]	Distributed PSO algorithm	Designed the jump-out strategy and revisit strategy
Chunying, W. et al. [25]	Adaptive vortex search (VS) Algorithm	Introduced an adaptive radius decrement process
Xinfang, L. et al. [26]	Shuffled frog-leaping algorithm (FLA)	Proposed a novel coding method and the worst frog update strategy

**Table 2:** Four tested functions.

Name	Definition	Domain	Optimum/Minimum
Ackley	$\begin{array}{l} {{}^{}}^{}f\left(x\right)=-20\exp\left(-0.2\sqrt{\sum_{i=1}^{D}\frac{x_{i}^{2}}{D}}\right)\\ {{}^{}}^{}-\exp\left(\sum_{i=1}^{D}\cos\left(2\pi x_{i}\right)/D\right)+20+e \end{array}$	[−32, −32]	[0, 0, 0, …., 0]/0
BentCigar	$\begin{array}{l} {{}^{}}^{}f\left(x\right)=x_{1}^{2}+10^{6}\sum_{i=2}^{D}x_{i}^{2} \end{array}$	[−100, 100]	[1, 1, 1, …, 1]/0
Schwefel	${{}^{}}^{}f(x)=418.9829*D-{\sum_{i=1}^{D}x_{i}\sin(\sqrt{\left|x_{i}\right|})}^{}{}^{}$	[−500, 500]	[420.96, 420.96, 420.96, ...., 420.96]/0
Weierstrass	$\begin{array}{l} f\left(x\right)=\sum_{n=0}^{+\infty }a^{n}\cos(b^{n}\pi x)\\ where\;0<a<1{,}^{}{}^{}ab>1+\frac{3}{2}\pi^{}{{}^{}}^{}{{}^{}}^{}{}^{} \end{array}$	[−50, 50]	[0, 0, 0, …, 0]/0

**Table 3:** Twelve benchmark functions.

Name	Definition	Domain	Optimum/Minimum
Sphere	$f_{1}\left(x\right)=\sum_{i=1}^{D}x_{i}^{2}$	[100, 100]	[0, 0, 0, …, 0]/0
Tablet	$f_{2}\left(x\right)={\left(1000*x_{1}\right)}^{2}+\sum_{i=2}^{D}x_{i}^{2}$	[−100, 100]	[0, 0, 0, …, 0]/0
StepFun	$f_{3}\left(x\right)={\sum_{i=1}^{D}\left(\left|x_{i}+0.5\right|\right)}^{2}$	[−100, 100]	[0, 0, 0, …, 0]/0
Rosenbrock	$f_{4}\left(x\right)={\sum_{i=1}^{D-1}\left(100\left(x_{i}^{2}-x_{i+1}\right)\right)}^{2}+{\left(x_{i}-1\right)}^{2}$	[−100, 100]	[1, 1, 1, …, 1]/0
Quadric	$f_{5}\left(x\right)={\sum_{i=1}^{D}\left(\sum_{j=1}^{i}x_{j}\right)}^{2}$	[−100, 100]	[0, 0, 0, …, 0]/0
BentCigar	$f_{6}\left(x\right)=x_{1}^{2}+10^{6}\sum_{i=2}^{D}x_{i}^{2}$	[−100, 100]	[1, 1, 1, …, 1]/0
Ackley	$\begin{array}{ll} f_{7}\left(x\right)= & -20\exp\left(-0.2\sqrt{\sum_{i=1}^{D}\frac{x_{i}^{2}}{D}}\right)\\ & -\exp\left(\sum_{i=1}^{D}\cos\left(2\pi x_{i}\right)/D\right)+20+e \end{array}$	[−32, −32]	[0, 0, 0, …., 0]/0
Griewank	$f_{8}\left(x\right)=\sum_{i=1}^{D}x_{i}^{2}/4000-\prod_{i=1}^{D}\cos\left(x_{i}/\sqrt{i}\right)+1$	[−600, −600]	[0, 0, 0, …, 0]/0
Rastrigrin	$f_{9}\left(x\right)=\sum_{i=1}^{D}\left(x_{i}^{2}-10\cos\left(2\pi x_{i}\right)+10\right)$	[−5.12, −5.12]	[0, 0, 0, …, 0]/0
RastrigrinNon	$\begin{array}{l} f_{10}\left(x\right)=\sum_{i=1}^{D}\left(y_{i}^{2}-10\cos\left(2\pi y_{i}\right)+10\right)\\ where\;y_{i}=\left\{\begin{array}{c} x_{i},\left|x_{i}\right|<1/2\\ \frac{round\left(2x_{i}\right)}{2},otherwise \end{array}\right. \end{array}$	[−5.12, −5.12]	[0, 0, 0, …, 0]/0
Penalized1	$f_{11}(x)=\sum_{i=1}^{D}u(x_{i},10,100,4)+\frac{\pi }{D}\left\{10(\sin{\left(\pi y_{1}\right)}^{2}\right.$ $+\sum_{i=1}^{D-1}({\left(y_{i}-1\right)}^{2}[1+10(\sin(\pi y_{i+1}))$ $\left.+{\left(y_{D}-1\right)}^{2}\right\}$	[−50, 50]	[1, 1, 1, …, 1]/0
Dminima	$f_{18}(x)=78.332331408+\sum_{i=1}^{D}\frac{x_{i}^{4}-16x_{i}^{2}+5x_{i}}{D}$	[−500, 500]	[420.96, 420.96, 420.96, ...., 420.96]/0

**Table 4:** Experimental results of benchmark functions.

Function	Algorithm	Best Value	Average Value	Standard Deviation
Sphere	CASSA	0	4.13 × 10^−4^	9.91 × 10^−3^
	WOA	1.51 × 10^−90^	6.88 × 10^2^	5.63 × 10^3^
	PSO	1.45 × 10^−3^	4.88 × 10^3^	8.45 × 10^4^
	ABC	3.16 × 10^−2^	8.18 × 10^2^	1.62 × 10^6^
	SSA	4.94 × 10^−299^	2.66 × 10^−5^	2.63 × 10^−4^
Tablet	CASSA	0	9.11 × 10^0^	1.93 × 10^2^
	WOA	2.03 × 10^−85^	2.36 × 10^4^	4.36 × 10^5^
	PSO	3.37 × 10^−1^	3.38 × 10^5^	1.69 × 10^6^
	ABC	1.45 × 10^−3^	5.76 × 10^4^	2.36 × 10^5^
	SSA	2.79 × 10^−54^	2.05 × 10^−15^	4.27 × 10^−15^
Stepfun	CASSA	2.08 × 10^−17^	1.03 × 10^−3^	8.87 × 10^−3^
	WOA	1.60 × 10^−2^	3.96 × 10^2^	3.90 × 10^3^
	PSO	1.91 × 10^2^	4.21 × 10^3^	1.33 × 10^4^
	ABC	3.91 × 10^3^	5.61 × 10^5^	3.51 × 10^6^
	SSA	4.03 × 10^−11^	1.66 × 10^−3^	2.68 × 10^−2^
Rosenbrock	CASSA	2.65 × 10^−9^	5.42 × 10^−2^	4.17 × 10^−1^
	WOA	2.78 × 10^1^	1.78 × 10^6^	1.61 × 10^7^
	PSO	7.44 × 10^2^	1.36 × 10^9^	4.93 × 10^8^
	ABC	5.34 × 10^2^	2.42 × 10^8^	6.16 × 10^9^
	SSA	2.94 × 10^−5^	6.64 × 10^−2^	4.17 × 10^−1^
Quadric	CASSA	0	1.40 × 10^−1^	3.08 × 10^2^
	WOA	0	8.82 × 10^−1^	2.62 × 10^−1^
	PSO	3.31 × 10^2^	1.46 × 10^4^	3.75 × 10^4^
	ABC	2.68 × 10^1^	7.11 × 10^3^	1.96 × 10^4^
	SSA	1.93 × 10^−135^	4.62 × 10^2^	2.31 × 10^2^
Bentcigar	CASSA	0	2.97 × 10^1^	4.01 × 10^2^
	WOA	8.59 × 10^−86^	7.47 × 10^8^	5.85 × 10^9^
	PSO	1.37 × 10^6^	1.47 × 10^6^	1.29 × 10^10^
	ABC	1.45 × 10^2^	1.05 × 10^0^	2.43 × 10^9^
	SSA	1.40 × 10^−156^	1.27 × 10^2^	1.75 × 10^3^
Ackley	CASSA	0	4.7 × 10^−03^	8.92 × 10^−2^
	WOA	3.55 × 10^−15^	5.92 × 10^−1^	2.79 × 10^0^
	PSO	5.55 × 10^0^	6.37 × 10^0^	2.75 × 10^0^
	ABC	3.16 × 10^−1^	3.67 × 10^0^	1.82 × 10^0^
	SSA	0	8.29 × 10^−4^	9.8 × 10^−3^
Griewank	CASSA	0	2.37 × 10^−5^	4.61 × 10^−4^
	WOA	0	5.40 × 10^0^	4.52 × 10^1^
	PSO	2.51 × 10^−5^	2.56 × 10^2^	1.91 × 10^2^
	ABC	3.63 × 10^−6^	6.65 × 10^4^	2.76 × 10^3^
	SSA	0	5.43 × 10^0^	4.57 × 10^1^
Rastrigrin	CASSA	0	4.35 × 10^−2^	9.70 × 10^−1^
	WOA	0	1.58 × 10^1^	5.92 × 10^1^
	PSO	7.95 × 10^−2^	7.55 × 10^+01^	2.47 × 10^1^
	ABC	3.83 × 10^−4^	4.60 × 10^0^	2.06 × 10^1^
	SSA	0	4.06 × 10^−1^	8.55 × 10^−1^
Rastrigrinnon	CASSA	0	2.97 × 10^−2^	6.12 × 10^−1^
	WOA	0	4.44 × 10^1^	7.89 × 10^1^
	PSO	7.41 × 10^−1^	7.52 × 10^1^	2.30 × 10^1^
	ABC	5.33 × 10^−3^	3.26 × 10^−01^	5.56 × 10^0^
	SSA	0	4.46 × 10^+01^	7.39 × 10^1^
Penalized1	CASSA	3.38 × 10^−19^	2.04 × 10^−6^	2.28 × 10^−5^
	WOA	1.27 × 10^−2^	3.38 × 10^6^	3.85 × 10^7^
	PSO	1.14 × 10^4^	1.17 × 10^4^	2.23 × 10^2^
	ABC	3.52 × 10^−2^	7.35 × 10^5^	1.46 × 10^6^
	SSA	1.56 × 10^−12^	7.43 × 10^−7^	6.21 × 10^−6^
Dminima	CASSA	0	2.04 × 10^−6^	2.28 × 10^−5^
	WOA	0	3.43 × 10^6^	3.85 × 10^7^
	PSO	2.80 × 10^2^	4.92 × 10^7^	7.63 × 10^6^
	ABC	1.92 × 10^−1^	3.52 × 10^3^	1.63 × 10^2^
	SSA	0	7.43 × 10^−7^	6.21 × 10^−6^

**Table 5:** Comparison of significance level results between CASSA and each algorithm.

Pair of Algorithms	*p*-Value
CASSA vs. WOA	1.9491 ×10^−4^
CASSA vs. PSO	5.9836 × 10^−5^
CASSA vs. ABC	2.4544 × 10^−4^
CASSA vs. SSA	7.9143 × 10^−3^

## Data Availability

Data sharing not applicable.

## References

[1] Huo L., Zhu J., Wu G., Li Z. (2020). A Novel Simulated Annealing Based Strategy for Balanced UAV Task Assignment and Path Planning. Sensors-Basel.

[2] Duchoň F., Babinec A., Kajan M., Beňo P., Florek M., Fico T., Jurišica L. (2014). Path Planning with Modified a Star Algorithm for a Mobile Robot. Procedia Eng..

[3] Sun Q.P., Li M., Wang T., Zhao C. UAV path planning based on Improved Rapidly-exploring Random Tree. Proceedings of the 30th Chinese Control and Decision Conference (2018 CCDC).

[4] Abeywickrama H.V., Jayawickrama B.A., He Y., Dutkiewicz E. Potential Field Based Inter-UAV Collision Avoidance Using Virtual Target Relocation. Proceedings of the 2018 IEEE 87th Vehicular Technology Conference (VTC Spring).

[5] Li L., Gu Q., Liu L. Research on Path Planning Algorithm for Multi-UAV Maritime Targets Search Based on Genetic Algorithm. Proceedings of the 2020 IEEE International Conference on Information Technology, Big Data and Artificial Intelligence (ICIBA 2020).

[6] Radmanesh M., Kumar M. (2016). Flight formation of UAVs in presence of moving obstacles using fast-dynamic mixed integer linear programming. Aerosp. Sci. Technol..

[7] Chithapuram C., Jeppu Y.V., Aswani Kumar C. Artificial Intelligence guidance for Unmanned Aerial Vehicles in three dimensional space. Proceedings of the 2014 International Conference on Contemporary Computing and Informatics (IC3I).

[8] Liu G., Peng B., Zhong X. (2021). Epidemic Analysis of Wireless Rechargeable Sensor Networks Based on an Attack–Defense Game Model. Sensors-Basel.

[9] Liu G., Peng B., Zhong X. (2021). A Novel Epidemic Model for Wireless Rechargeable Sensor Network Security. Sensors-Basel.

[10] Zhang D., Duan H. (2018). Social-class pigeon-inspired optimization and time stamp segmentation for multi-UAV cooperative path planning. Neurocomputing.

[11] Shin J., Bang H. (2020). UAV Path Planning under Dynamic Threats Using an Improved PSO Algorithm. Int. J. Aerosp. Eng..

[12] Cekmez U., Ozsiginan M., Sahingoz O.K. Multi colony ant optimization for UAV path planning with obstacle avoidance. Proceedings of the 2016 International Conference on Unmanned Aircraft Systems (ICUAS).

[13] Li B., Gong L., Yang W. (2014). An Improved Artificial Bee Colony Algorithm Based on Balance-Evolution Strategy for Unmanned Combat Aerial Vehicle Path Planning. Sci. World J..

[14] Pan J., Liu J., Hsiung S. Chaotic Cuckoo Search Algorithm for Solving Unmanned Combat Aerial Vehicle Path Planning Problems. Proceedings of the 2019 Association for Computing Machinery.

[15] Pandey P., Shukla A., Tiwari R. (2018). Three-dimensional path planning for unmanned aerial vehicles using glowworm swarm optimization algorithm. Int. J. Syst. Assur. Eng. Manag..

[16] Chen Y.B., Mei Y.S., Yang J.Q., Sun X.L. (2017). Three-dimensional unmanned aerial vehicle path planning using modified wolf pack search algorithm. Neurocomputing.

[17] Wang G., Chu H.E., Mirjalili S. (2016). Three-dimensional path planning for UCAV using an improved bat algorithm. Aerosp. Sci. Technol..

[18] Wu J., Wang H., Li N., Yao P., Huang Y., Yang H. (2018). Path planning for solar-powered UAV in urban environment. Neurocomputing.

[19] Qu C., Gai W., Zhang J., Zhong M. (2020). A novel hybrid grey wolf optimizer algorithm for unmanned aerial vehicle (UAV) path planning. Knowl. Based Syst..

[20] Li J. A modified particle swarm optimization based on genetic algorithm and chaos. Proceedings of the 11th World Congress on Intelligent Control and Automation.

[21] Pierre D.M., Zakaria N., Pal A.J. Master-Slave Parallel Vector-Evaluated Genetic Algorithm for Unmanned Aerial Vehicle’s path planning. Proceedings of the International Conference on Hybrid Intelligent Systems.

[22] Liu G., Wang X., Liu B., Wei C., Li J. Path Planning for Multi-Rotors UAVs Formation Based on Ant Colony Algorithm. Proceedings of the 2019 International Conference on Intelligent Computing, Automation and Systems (ICICAS).

[23] Sun Y., Chen J., Du C., Gu Q. Path planning of UAVs based on improved Clustering Algorithm and Ant Colony System Algorithm. Proceedings of the 2020 IEEE 5th Information Technology and Mechatronics Engineering Conference (ITOEC 2020).

[24] Wang Y., Bai P., Liang X., Wang W., Zhang J., Fu Q. (2019). Reconnaissance Mission Conducted by UAV Swarms Based on Distributed PSO Path Planning Algorithms. IEEE Access.

[25] Wang C., Liu P., Zhang T., Sun J. The Adaptive Vortex Search Algorithm of Optimal Path Planning for Forest Fire Rescue UAV. Proceedings of the 2018 IEEE 3rd Advanced Information Technology, Electronic and Automation Control Conference (IAEAC 2018).

[26] Li X., Fang Y., Fu W. UAV Path Planning Based on Shuffled Frog-Leaping Algorithm and Dubins Path. Proceedings of the 39th Chinese Control Conference.

[27] Xue J., Shen B. (2020). A novel swarm intelligence optimization approach: Sparrow search algorithm. Syst. Sci. Control Eng..

[28] Kumaravel S., Ponnusamy V. (2020). An Efficient Hybrid Technique for Power Flow Management in Smart Grid with Renewable Energy Resources. Energy Sources Part A Recovery Util. Environ. Eff..

[29] Liu B., Rodriguez D. (2021). Renewable energy systems optimization by a new multi-objective optimization technique: A residential building. J. Build. Eng..

[30] Wang Y., Wang H. Obstacle Avoidance of UAV Based on Neural Networks and Interfered Fluid Dynamical System. Proceedings of the 2020 3rd International Conference on Unmanned Systems (ICUS).

[31] Zhu Y., Yousefi N. (2021). Optimal parameter identification of PEMFC stacks using Adaptive Sparrow Search Algorithm. Int. J. Hydrog. Energy.

[32] Wang Y.L. Image Scrambling Method Based on Chaotic Sequences and Mapping. Proceedings of the 2009 First International Workshop on Education Technology and Computer Science.

[33] Gálvez J., Cuevas E., Becerra H., Avalos O. (2020). A hybrid optimization approach based on clustering and chaotic sequences. Int. J. Mach. Learn. Cybern..

[34] Artiles J.A.P., Chaves D.P.B., Pimentel C. (2019). Image encryption using block cipher and chaotic sequences. Signal Process. Image Commun..

[35] Ksheerasagar T.K., Anuradha S., Avadhootha G., Sai Ram Charan K.V.S.D., Sri Hari Rao P. Performance analysis of DS-CDMA using different chaotic sequences. Proceedings of the 2016 International Conference on Wireless Communications, Signal Processing and Networking (WiSPNET).

[36] Li C., Zhang N., Lai X., Zhou J., Xu Y. (2017). Design of a fractional-order PID controller for a pumped storage unit using a gravitational search algorithm based on the Cauchy and Gaussian mutation. Inf. Sci..

[37] Ebraheem M., Jyothsna T.R. Comparative performance evaluation of teaching learning based optimization against genetic algorithm on benchmark functions. Proceedings of the 2015 IEEE Power, Communication and Information Technology Conference (PCITC).

[38] Kochengin A.E., Chrysostomou G., Shikhin V.A. Performance of Nonparametric Wilcoxon Test with Reference to the Samples with Singularities. Proceedings of the 2019 III International Conference on Control in Technical Systems (CTS).

[39] Ammad M., Ramli A. Cubic B-Spline Curve Interpolation with Arbitrary Derivatives on its Data Points. Proceedings of the 2019 23rd International Conference in Information Visualization—Part II.

[40] Li Q.S., Li J.P. A Bezier Curve-Based Font Generation Algorithm for Character Fonts. Proceedings of the IEEE 16th International Conference on Smart City.

[41] Liu Z., Tan J., Chen X., Zhang L. (2012). An approximation method to circular arcs. Appl. Math. Comput..

[42] Causa F., Fasano G., Grassi M. (2018). Multi-UAV Path Planning for Autonomous Missions in Mixed GNSS Coverage Scenarios. Sensors-Basel.

